# Self-Healable Biocomposites Crosslinked with a Combination of Silica and Quercetin

**DOI:** 10.3390/ma14144028

**Published:** 2021-07-19

**Authors:** Olga Olejnik, Anna Masek, Małgorzata Iwona Szynkowska-Jóźwik

**Affiliations:** 1 Institute of Polymer and Dye Technology, Faculty of Chemistry, Lodz University of Technology, Stefanowskiego 12/16, 90-924 Lodz, Poland; olejnik.olga@dokt.p.lodz.pl; 2 Institute of General and Ecological Chemistry, Faculty of Chemistry, Lodz University of Technology, Żeromskiego 116, 90-924 Lodz, Poland; malgorzata.szynkowska@p.lodz.pl

**Keywords:** epoxidized natural rubber, ENR-50, quercetin, bio-based composite, natural crosslinker, self-healable, self-healing, biocomposite

## Abstract

In this publication, novel bio-based composites made of epoxidized natural rubber with 50 mol% of epoxidation (ENR-50) are presented. The obtained materials, partially cured with a totally environmentally friendly crosslinking system consisting of natural ingredients, including quercetin and silica, exhibit a self-healing ability resulting from the self-adhesion of ENR-50 and reversible physical forces between the curing agent and the matrix. The impact of natural components on the crosslinking effect in uncured ENR-50 matrix was analyzed based on rheometric measurements, mechanical tests and crosslinking density. The partially crosslinked samples were next cut into two separate pieces, which were instantly contacted together under a small manual press, left at room temperature for a few days for the healing process to occur and finally retested. The healing efficiency was estimated by measuring mechanical properties before and after the healing process and was also confirmed by photos taken using optical and scanning electron microscope (SEM). According to the results, a combination of silica and quercetin is a totally safe, natural and effective crosslinking system dedicated to epoxidized natural rubber. The novel composites containing ingredients safe for human beings exhibit promising self-healing properties with a healing efficiency of up to 45% without any external stimuli and stand a chance of becoming innovative biomedical materials.

## 1. Introduction

From the ecological point of view, epoxidized natural rubber (ENR) is a very attractive material. Because of its origin, it is characterized by a negative carbon footprint and therefore qualifies as an environmentally friendly elastomer that can be employed in biocomposite preparation [1]. This material is produced by means of epoxidation process, i.e., controlled chemical modification of natural rubber (NR), which is a popular biopolymer derived from the *Hevea brasiliensis* plant [2]. However, epoxidized natural rubber has a much more interesting structure as well as improved properties in comparison to its precursor [3,4].

The chain of ENR consists of two types of functional moieties, namely oxirane rings and a double bonding; thus, such a polymer has more possible ways to be cured than natural rubber or conventional synthetic elastomers [5]. On the one hand, double bonds enable ENR to be cured using classic vulcanization systems, including sulfur assisted with activators and accelerators, where sulfidic crosslinks between the rubber molecules are formed, or by utilizing dicumyl peroxide (DCP) and creating carbon–carbon crosslinks between the rubber chains [6]. On the other hand, reactive oxirane groups present in the structure allow curing by multifunctional nucleophilic reagents, including amines most effectively in combination with phenol or bisphenol A as a catalyst [7], and by carboxylic acids with imidazole as an accelerator [8], as well as by anhydrides [9]. It is assumed that also hydroxyl groups of silica [10] or -OH moieties derived from phenolic compounds, including lignin [7], can react with oxirane groups in ENR and form new bonds between chains. Undoubtedly, more functional groups present in the structure give more possible ways to crosslink, also by using only safe and natural ingredients, which may play a key role in composite applications in many fields, including the medical field.

Unlike natural rubber (NR), epoxidized natural rubber (ENR) due to highly polar epoxide groups not only has more possible ways for crosslinking but also is characterized by improved properties, such as better oil and nonpolar solvent resistance [11]. Moreover, ENR is characterized by good adhesion to wet surfaces and low rolling resistance, as well as low gas permeability [10,11]. Furthermore, this elastomer, with maximally 50 mol% of epoxidation, is resistant to crack propagation, because it crystallizes under strain and is able to reach high elongation at break [8,12,13]. It must be also mentioned that the properties of ENR considerably depend on epoxidation degree; for example, the higher the level of epoxidation, the higher the glass transition temperature (T_g_) [8]. Interestingly, epoxidized natural rubber also exhibits self-healing ability manifested in the self-adhesion phenomenon [14]. It was found that the chain relaxation process plays a key role in the self-healing and adhesive properties of partially cured ENR. The interpenetration of free chain ends is possible thanks to molecular mobility. Moreover, epoxide ring-opening during the ENR synthesis causes the formation of hydroxyl and carboxyl groups, which also contributes to the self-healing phenomenon occurrence through hydrogen bond appearance at the interface of rubber joints [15].

Self-healing properties of epoxidized natural rubber (ENR) were firstly detected by Rahman et al. [16] in blends based on ethylene/methacrylic acid copolymer ionomer with ENR by conducting a ballistic puncture test. Subsequently, a self-repairing behavior of macromolecular chain (ENR) and ethylene methacrylic acid groups partially neutralized with metal ions was also studied by the same group [17]. In addition, these scientists analyzed the self-healing ability of pure epoxidized natural rubber [14], which contributed to the appearance of novel self-healable composites. Over the last decade, the number of self-repairable composites based on ENR has grown [18,19,20,21,22,23]. Such materials can reveal self-healing ability under external stimuli, including increased temperature, where exchanging reactions are conducted [24,25,26,27]. On the other hand, some ENR-based composites are able to self-repair without any stimulus [28].

The proper selection of natural and safe additives dedicated to epoxidized natural rubber may result in obtaining totally environmentally friendly self-healable biocomposites. One of the most interesting bio-based material with self-repairing ability was designed by Cao et al. [15]. The authors prepared a self-healable epoxidized natural rubber composite filled with cellulose nanocrystals isolated from marine biomass, and the material was slightly cured using dicumyl peroxide (DCP). Another study worth mentioning research is that of Xu et al. [29], who prepared recyclable and heat-healable biocomposites made of ENR containing an environmentally friendly crosslinker, i.e., citric acid modified bentonite (CABt). The same scientific group also created self-healable epoxidized natural rubber/carboxymethyl chitosan bio-based composites with improved mechanical properties and self-healing efficiency of 90% at room temperature [30]. The high healing efficiency at ambient temperature without any external stimuli was also achieved by Nie et al. [28], who made bio-based composites of ENR and chitin nanocrystals containing a hydrogen bonding supramolecular network. Nevertheless, these composites had a low tensile strength of about 1.19 MPa and therefore were next filled with carbon nanotubes [31]. The bio-based and self-healable composites crosslinked and filled with totally safe substances have a chance of being applied in many fields, including biomedical and dental branches.

In our research, to obtain self-healable biocomposites, it was proposed to reduce crosslinking density in ENR-50 by replacing conventional curing agents, such as dicumyl peroxide (DCP), with a combination of more pro-ecological substances, i.e., quercetin and silica. A popular natural antioxidant—quercetin—was responsible for the low crosslinking density of ENR-50, and silica filler provided enhanced mechanical properties. In the studied self-healing phenomenon, which occurs in ambient conditions, physical forces play a key role. The relationship between the self-healing ability and mechanical properties of prepared materials was studied. Such composites stand a chance of becoming innovative self-repairable biomaterials.

Because of the good adhesive properties of ENR-50 and oral cavity-safe ingredients [32,33,34], such composites have the potential to be dedicated to self-healing long-term soft denture lining materials production [35]. However, more advanced, especially from the medical point of view, studies must be carried out in the future to confirm our presumptions.

## 2. Materials and Methods

### 2.1. Materials and Processing

The tested composites were made of ENR rubber with 50 mol% epoxidation produced by Muang Mai Guthrie Company Limited (Phuket, Thailand) under the name of Dynathai Epoxyprene 50 (ENR-50). Quercetin hydrate (≥95% of purity) obtained from Sigma Aldrich (Munich, Germany) was applied as an alternative crosslinker. To improve the curing effect and mechanical properties, hydrophilic fumed silica (Aerosil 380) with a specific surface area of 380 m^2^/g purchased from Evonik Operations GmbH (Essen, Germany) was used. Moreover, dicumyl peroxide (DCP, bis(α,α-dimethylbenzyl)peroxide) (98% of purity) obtained from Merck (Darmstadt, Germany) was utilized as a traditional crosslinker in the referential sample.

The studied materials were prepared in accordance with the compositions presented in Table 1.

Firstly, the components were mixed in a laboratory micromixer (Brabender Lab-Station purchased from Plasti-Corder and equipped with Julabo cooling system (Duisburg, Germany)). The mixing process was conducted for 15 min at ambient temperature with the speed of 60 rpm. The temperature spontaneously rose to around 70 °C during the operation. Subsequently, the prepared materials were formed using a laboratory mixing mill with a friction of 1–1.2 at room temperature. After analyzing the curing effect of the prepared mixtures, the materials were finally vulcanized for 20 min at the temperature of 160 °C and the pressure of 14 MPa using an electrically heated hydraulic laboratory press (Skamet 54436, SKAMET, Skarzysko-Kamienna, Poland). Thanks to the application of special steel vulcanization molds situated between the press shelves, the obtained rectangular samples were 120 mm long, 80 mm wide and around 1 mm thick. Moreover, polytetrafluoroethylene (PTFE, Teflon) films purchased from Holtex^®^ (Rzgow, Poland) were applied as spacers to prevent the adherence phenomenon. The prepared samples were analyzed using the different methods described below.

### 2.2. Rheometric Study

The prepared unvulcanized materials were analyzed in terms of their curing effect using an Alpha MDR 2000 oscillating disc rheometer (Alpha Technologies, Hudson, OH, USA). The measurement was conducted at the temperature of 160 °C for 60 min. The obtained results, including maximal torque ($M_{max}$) and minimal torque ($M_{min}$) were applied for calculating an increase in torque as $\Delta M=M_{max}-M_{min}$. Because of the fact that a torque increase is related to the degree of crosslinking, we could choose the best curing time for obtaining slightly cured composites, which reveal better mechanical properties than uncured pure epoxidized natural rubber and satisfactory self-healing ability. Therefore, the materials were next compressed for 20 min. The increase in torque after 20 min ($\Delta M_{20}$) was also calculated as $\Delta M_{20}=M_{20}-M_{min}$, where $M_{20}$ is torque after 20 min of curing.

### 2.3. Crosslink Density Analysis

To determine the crosslink density, samples of around 0.03–0.05 g were swollen to equilibrium for 72 h at room temperature and weighed. Then, the same samples were dried for about 96 h at T = 40 °C to constant mass in a Series FD Binder dryer with circulated air (Binder, Tuttlingen, Germany) and weighed again. The crosslink density was calculated according to the Flory–Rehner Equation (1) [36,37]:

(1) $$v=-\frac{\ln\left(1-V_{r}\right)+V_{r}+\mu V_{r}^{2}}{V_{0}\left(V_{r}^{\frac{1}{3}}-\frac{2V_{r}}{f}\right)}$$

where v  is the crosslink density (mol/cm^3^) $V_{r}$ is the volume fraction of rubber in a swollen sample (-), $V_{0}\;$ is the solvent molar volume (cm^3^/mol) (for toluene, V_0_ = 106.2 cm^3^/mol [18]), f is the functionality of crosslink (-) (f = 4, assuming the formation of tetra-functional crosslink) and μ is the Flory–Huggins rubber–solvent interaction parameter (for the investigated ENR50–toluene system, *μ* = 0.42 [18]).

The given crosslink density values are the average of four specimens of every sample. The relative standard deviation is approximately 5.0% on average.

### 2.4. Mechanical Properties and Self-Healing Ability Investigation

**Figure 1:**
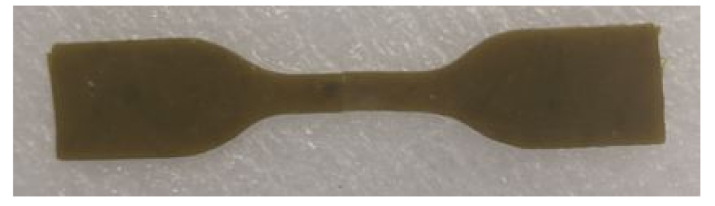
Exemplary ENR/quercetin2/silica15 sample (type 2 according to ISO 37 standard) dedicated to mechanical test.

Partially cured plates of ENR-50-based materials were cut into dumbbell-shaped samples type 2 according to PN-ISO 37:1998 standard [38] for smaller preferred size (Figure 1), which are approximately 1 mm thick, 75 mm long and 12.5 mm wide at ends. Five dumbbell-shaped specimens type 2, of every prepared material, were tested in terms of mechanical properties using the universal mechanical testing machine Zwick 1435 (Zwick Roell GmbH & Co. KG, Ulm, Germany) equipped with an extensometer. The measurement was carried out for prepared samples at a crosshead speed of 500 mm/min according to PN-ISO 37:1998 standard.

The rest of the samples were each cut into two separate pieces from their middle section. Subsequently, the two fresh surfaces were instantly connected together under a small manual press and left on a table in order to heal at room temperature for 2 days (first group), for 4 days (second group) and for 4 days with 30 min of heating at 160 °C in FD Series Binder dryer with circulated air (Binder, Tuttlingen, Germany) (third group). The cut specimens after the selected period were tested using the same mechanical testing machine. The obtained results, including tensile strength (TS) and elongation at break (Eb), were compared with pristine samples. Moreover, the healing efficiency (R) was calculated in two ways in accordance with the following Equations (2) and (3), where tensile strength and elongation at break are analyzed:

(2) $$R_{TS}\;\left[\%\right]=\frac{TS_{after\;healing}}{TS_{before\;healing}}*100\%$$

where *R_TS_* (%) is the healing efficiency from tensile strength (TS) (MPa), *TS_after healing_* (MPa) is the tensile strength of healed material and *TS_before healing_* (MPa) is the tensile strength of pristine material.

(3) $$R_{Eb}\;\left[\%\right]=\frac{Eb_{after\;healing}}{Eb_{before\;healing}}*100\%$$

where *R_Eb_* (%) is healing efficiency from elongation at break (Eb) (%), *Eb_after healing_* (MPa) is the elongation at break of healed material and *Eb_before healing_* (MPa) is the elongation at break of pristine material.

### 2.5. Fourier Transform Infrared Spectroscopy (FT-IR) Absorbance Spectra Analysis

The chemical structure of the prepared ENR-50-based composites was characterized using Thermo Scientific Nicolet 6700 Fourier transform infrared spectroscopy (FT-IR) spectrometer with diamond Smart Orbit ATR sampling equipment (Thermo Fischer Scientific Instruments, Waltham, MA, USA). The spectra obtained in the absorption mode were investigated in the range of 4000–400 cm^−1^ with the use of 64 scans and resolution of 4 cm^−1^.

### 2.6. Microscopic Observation of Healing Effect

The surfaces of pristine and healed composites were observed using Leica MZ6 stereoscopic microscope (Heerbrugg, Switzerland) with MultiScan 8.0 image analysis software (CSS, Warsaw, Poland) at a magnification of 50×. Additional photos were taken using an S-4700 scanning electron microscope (Hitachi, Tokyo, Japan), equipped with an energy dispersive spectrometer (SEM-EDS) (ThermoNoran, Madison, WI, USA).

## 3. Results and Discussion

### 3.1. Rheometric Study

**Figure 2:**
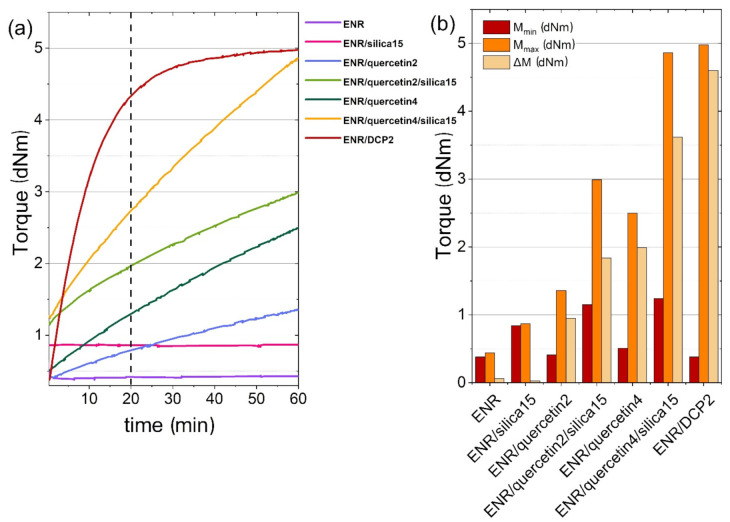
Analysis of curing effectiveness of ENR-50-based materials in a function of time (**a**) and values of the minimal torque (M_min_), maximal torque (M_max_) and torque increase (ΔM) of the same materials (**b**).

The curing effect of the prepared composites was investigated using an oscillating disc rheometer. The obtained rheometric curves are presented in Figure 2a and torque values depending on the composition are shown in Figure 2b.

The ENR-50-based materials containing a novel bio-based crosslinking agent, i.e., quercetin or a combination of quercetin and silica, revealed a curing effect at the temperature of 160 °C, which can be observed as increasing torque in a function of time. Interestingly, implementing 15 phr of only silica with no quercetin addition caused only growth in minimal torque but did not result in the growth of maximal torque as well as in an increase in changing the torque during the test. The difference between the minimal torques of pure ENR-50 and ENR-50 filled with silica can be a result of interaction between the polymer matrix and a filler, where hydrogen bonds are likely to form. Xu et al. [10] verified that a higher amount of silica is required for higher torque increase and higher minimal torque, which can also correspond with crosslink density as well a better crosslinking effect. This phenomenon was explained by increased contact areas of epoxy groups and Si-OH as a result of the higher amount of silica. Nevertheless, it was confirmed that hydroxyl groups located in silica molecules also contribute to the curing of ENR, and hydrogen bonds are of great importance in this process. The low values of maximal torque and change in torque in comparison to other systems can be a result of a too low amount of silica addition. Therefore too few bonds and only unstable crosslinks and weak forces between Si-OH groups of silica aggregates and oxirane rings of ENR were formed, which appear and disappear at the same time during a rheometric measurement. Contrary to ENR/silica15, pure ENR is not a self-crosslinkable material and the crosslinks do not appear at all. Adding only 2 phr of quercetin to pure epoxidized natural rubber and to ENR filled with silica caused a visible rise in torque in a function of time of about 0.9 dNm in the case of ENR/quercetin2 and of about 1.8 dNm in the case of ENR/quercetin2/silica15. Such a phenomenon can be explained by the presence of phenolic functional groups in the structure of quercetin. Such groups can react with oxirane rings and also contribute to the opening of these rings. A similar effect was detected by Jiang et al. [7], where epoxidized natural rubber was mixed with lignin, which also contains hydroxyl groups in its structure. The higher the amount of lignin was, the faster the torque increased in a function of time. The addition of a higher amount of quercetin also makes the curing process more effective, especially in a material filled with silica. The sample containing a combination of 4 phr of quercetin and 15 phr of silica reached a maximal torque of 4.86 dNm, which was similar to the M_max_ of the referential sample: 4.98 dNm. The torque increase (ΔM) also was the closest to the results of the referential sample (ΔM = 4.60 dNm) from all studied materials and amounted to 3.62 dNm. Moreover, the curing effect of ENR/quercetin2/silica15 material appearing in torque increase is a bit lower than the result of ENR/quercetin4. It could be assumed that the addition of silica is unnecessary, but associating these results with the mechanical properties explains the need for silica presence. To obtain partially crosslinked composites, the prepared materials were vulcanized for 20 min. The torque results obtained after 20 min of composite vulcanization are introduced in Table 2.

Such results differ from the values obtained after 60 min of measurement. According to torque increase values received after 20 min, the lowest crosslinking effect is observed in pure ENR and ENR with 15 phr of silica. These phenomena occur because the crosslinks are not formed during the heating or are not stable. On the other hand, taking into account the ENR–silica structure possibly formed during processing, which is represented by torque values obtained at the 20th min of vulcanization, the lowest crosslinking effect is visible in pure ENR and ENR containing 2 phr of quercetin.

### 3.2. Crosslink Density

**Figure 3:**
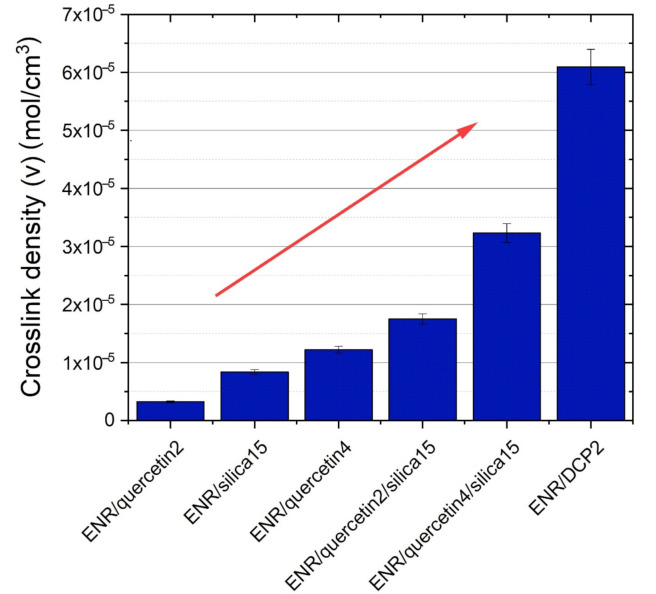
Crosslink density of partially cured ENR-50-based composites.

According to Figure 3, the crosslink density values correspond to torque results in the 20th min of rheometric measurement. In spite of the fact that the torque increase (ΔM) of ENR/silica15 composite is almost imperceptible, the high minimal torque (M_min_) and torque in the 20th min (M_20_) indicates that some interactions between the components exists in this composite. This can be explained by crosslink formation during processing, where oxirane rings are able to open at mildly acidic conditions [5], and then new crosslinks are formed and reversible hydrogen bonds are created. It must be underlined that added silica Aerosil 380 provides gently acidic conditions and therefore facilitates the opening of oxirane rings. During the 20th min of vulcanization, the referential sample (ENR/DCP2) composite reached the highest crosslinking density of about 6.1 × 10^−5^ mol/cm^3^.

### 3.3. Mechanical Properties and Self-Healing Ability Investigation

**Figure 4:**
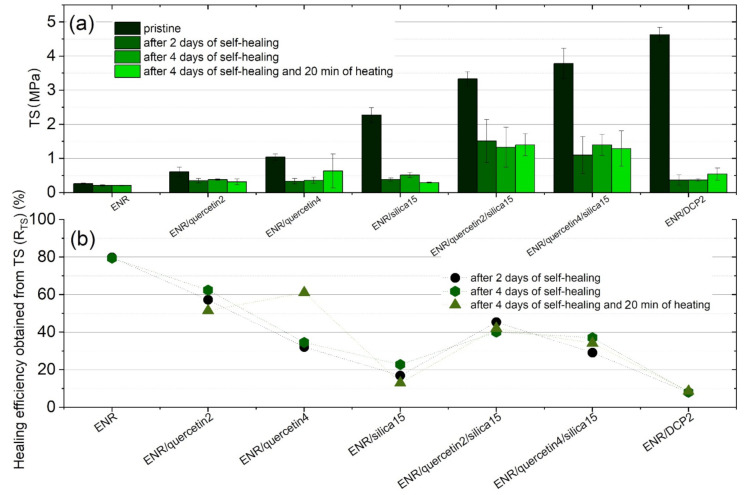
Tensile strength (TS) of tested materials before and after the self-healing process (**a**) and healing efficiency calculated using TS results for every material after 2 days, 4 days and 4 days with 20 min of heating (**b**).

Tensile strength (TS) results, which correspond to the healing efficiency (R_TS_) values of tested materials, are introduced in Figure 4a,b and also in Tables S1 and S2. As can be noticed, uncured and slightly cured ENR have the highest healing efficiencies of about 80%. Such high healing efficiency of pure ENR has also been investigated by Rahman et al. [14] and has been explained as a self-adhesion effect of damaged surfaces. Nevertheless, uncured epoxidized natural rubber is characterized by poor mechanical properties, including tensile strength; therefore, the curing process is an indispensable element for this material. On the other hand, ENR effectively cured with dicumyl peroxide revealed poor healing efficiency of about 8%. It can be assumed that when ENR-50-based material has the highest mechanical properties, it reveals the lowest healing efficiency, and a compromise between these abilities must be found. Nevertheless, such a tendency is noticed only for ENR cured with pure quercetin or pure silica. A surprising effect was obtained after adding a combination of quercetin and silica to pure ENR. These composites are characterized by tensile strengths of 3.3 ± 0.2 MPa for ENR/quercetin2/silica15 sample and 3.8 ± 0.4 MPa for ENR/quercetin4/silica15 material. Such results are visibly higher than for ENR containing only silica, which is characterized by TS of 2.3 ± 0.2 MPa. Interestingly, such composites also have a better healing efficiency, which amounted to around 40–45% for ENR/quercetin2/silica15 and approximately 30–37% for ENR/quercetin4/silica15, depending on time healing and additional factors, including increased temperature. A combination of hydroxyl groups derived from silica and multiple phenolic groups belonging to quercetin, which are present in ENR/quercetin/silica, triggered not only satisfying mechanical properties but also a promising healing efficiency.

**Figure 5:**
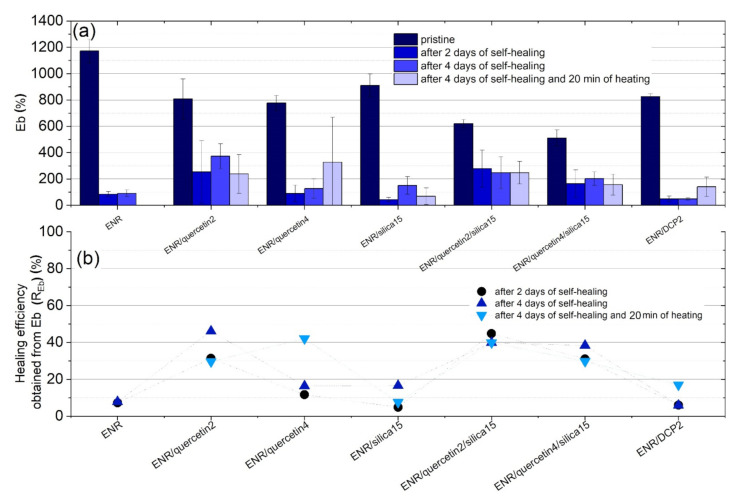
Elongation at break (Eb) of tested materials before and after self-healing process (**a**) and healing efficiency calculated using Eb results for every material after 2 days, 4 days and 4 days with 20 min of heating (**b**).

Restoration of the damaged material should be analogously visible in the regaining of tensile strength but should also reveal the recovery of other properties, such as elongation at break (Eb). The healing efficiency can be analogously calculated using the results of Eb. The results of elongation at break and calculated healing efficiency values (R_Eb_) are presented in Figure 5a,b. Uncured epoxidized natural rubber is characterized by high elongation at break of above 1200%. Nevertheless, this material is not able to regain this value after the healing process. Moreover, the time of healing did not influence the healing effect (R_Eb_) significantly, which amounted to around 8%. Furthermore, heating this material caused sample destruction. On the other hand, the samples of conventionally crosslinked ENR-50 using DCP were not able to regain Eb values effectively, and their healing efficiency amounted to approximately 6%. Nevertheless, the heating of ENR/DCP2 caused an increase in this value to 16%. The heating process was also advantageous for the ENR/quercetin2 sample. For other materials, heating caused a deterioration in healing efficiency (R_Eb_). Partially cured ENR-50 using a combination of quercetin and silica revealed relatively high healing efficiency (R_Eb_), which equaled approximately 40–45% for ENR/quercetin2/silica15 and around 30–38% for ENR/quercetin4/silica15. These healing efficiency results calculated from Eb results of ENR-50 crosslinked with a combination of quercetin and silica correspond with healing efficiency values obtained from TS; therefore, the complex repair ability has been acquired in comparison to the other studied materials.

Based on mechanical test results, the uncured ENR-50, similarly to other typical unvulcanized rubbers, is characterized by low tensile strength (TS), and thus the crosslinker is an indispensable part of such elastomeric composites to make ENR-50 useful. The crosslinking process using only silica15 or DCP2 resulted in a significant increase in strength and a slight decrease in elongation of ENR-50 and deterioration in self-healing ability. On the other hand, ENR-50 samples with quercetin became more rigid because aromatic rings, which are present in the structure, have better self-healing ability but need a filler to improve the strength of the materials. The ENR-50-based composites containing a combination of quercetin and silica are stronger but also stiffer than the samples cured using only quercetin or only silica because of the strong connection between quercetin molecules and silica aggregates. Such materials reveal satisfactory self-healing properties despite silica presence. Moreover, elongation at break of pristine ENR/quercetin/silica composites is around 200% lower than that of highly cured ENR/DCP material, which is also caused by the presence of rigid aromatic structures belonging to quercetin. Furthermore, the ENR/quercetin4/silica15 composite is characterized by higher elongation at break (Eb) in comparison to ENR/quercetin2/silica15. A higher amount of quercetin, containing an aromatic rings in its structure, causes an increase in the rigidity of the material. Moreover, more hydroxyl groups entail more crosslinks; thus an increase in tensile strength and a decrease in elongation at break occur.

### 3.4. Fourier Transform Infrared Spectroscopy (FT-IR) Absorbance Spectra Analysis

**Figure 6:**
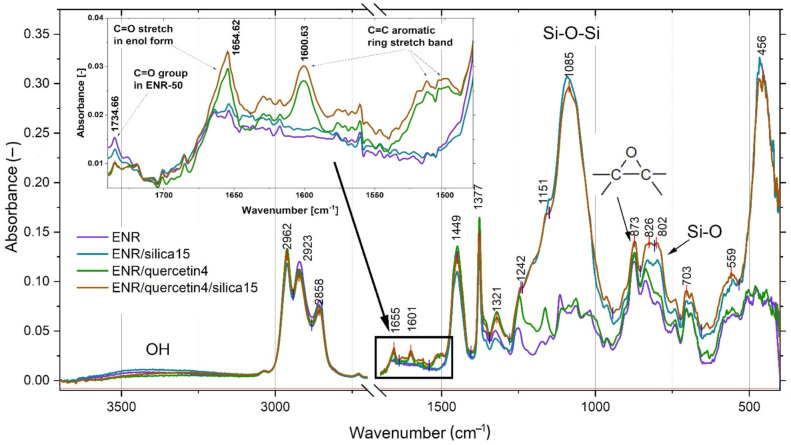
FT-IR spectrum of ENR/quercetin4/silica15 compared with spectra of pure ENR, ENR/silica15 and ENR/quercetin4.

The obtained spectra of ENR/quercetin/silica composites were compared with the spectrum of pure ENR, the spectrum of ENR composite crosslinked with quercetin and the spectrum of ENR material cured with silica. The characteristic spectra are presented in Figure 6.

**Figure 7:**
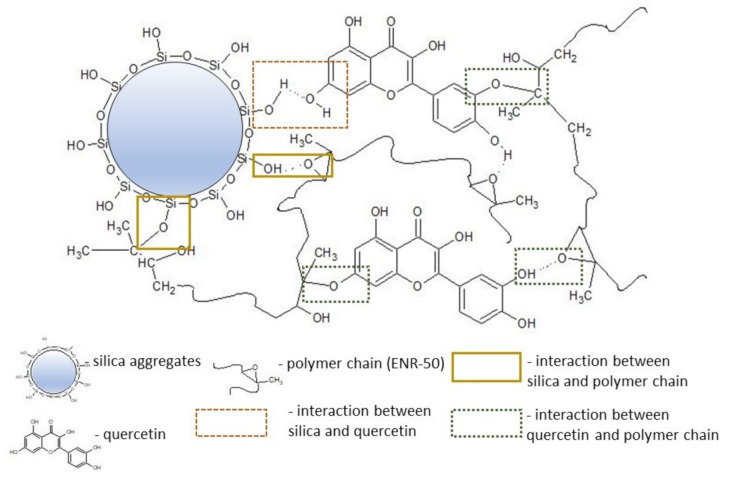
Proposed structure and interactions in ENR/quercetin/silica composite.

The elastomeric composite consisting of ENR, quercetin and silica, similarly to pure ENR, ENR with pure silica and ENR with pure quercetin, is characterized by chemical moieties, including methyl asymmetrical stretching vibration (2962 cm^−1^), methyl asymmetrical deformation vibration (1449 cm^−1^) and methyl symmetrical deformation vibration (1377 cm^−1^), which were also detected by Xu et al. [39]. The observed bands at 2923 and 2858 cm^−1^ correspond to the asymmetrical stretching vibration of methylene group and the symmetrical stretching vibration of methylene group respectively. The characteristic peak of asymmetrical stretching vibration of epoxy ring is visible at 873 cm^−1^, but another specific band for this moiety, which is visible in pure ENR material, is connected to a stronger band of Si-O at about 795–802 cm^−1^ in ENR/quercetin/silica composite. Moreover, Catauro et al. [40], studying hydrogen bond interactions between quercetin and silica in silica/quercetin sol–gel hybrids dedicated to dental implant materials, assumed that wavenumbers at 820 and 793 cm^−1^ belong to aromatic out-of-plane C-H bending vibrations. The convergence of the presented groups caused the appearance of a specific band at about 802–826 cm^−1^. Rahman et al. [14] mentioned the presence of cyclic ether at 1070 cm^−1^, but in ENR/quercetin/silica composites, such a band is invisible in spectra because of strong Si-O-Si asymmetrical stretching vibrations at 1085 cm^−1^ [40]. In the spectrum of ENR/quercetin/silica composites, specific peaks at 1600 and 1654 cm^−1^ are visible. According to Catauro et al., the wavenumber of 1610 cm^−1^ corresponds to the C=C aromatic ring stretching band. Another characteristic peak at about 1654 cm^−1^ probably belongs to C=O stretching in enol form, which was confirmed by Kannan et al. [41] in their analysis of seagrass polyphenols. The important C-O groups in ENR/quercetin/silica and ENR/quercetin composites are identified at 1321 cm^−1^ [42] and are possibly responsible for the permanent interaction between hydroxyl groups of quercetin molecules and the chains of epoxidized natural rubber. The most important chemical groups in the tested composites are -OH groups, which are visible in spectra at 3200–3450 cm^−1^, and such groups probably significantly participate in the self-healing process of the tested ENR/quercetin/silica composites. Such bonds are reversible and are able to promote the self-adhesion process in the ENR matrix. The proposed structure of the ENR/quercetin/silica composite is presented in Figure 7. As can be noticed, the hydroxyl groups of quercetin and silica and the oxirane rings in the ENR chain play a key role in creating stable crosslinks. Hydroxyl groups of silica or quercetin react with epoxy groups of ENR. The same hydroxyl groups are also responsible for creating physical interactions, including hydrogen bonding, not only between silica and ENR or between quercetin and ENR but also between silica and quercetin. Such interactions possibly also play a key role in the self-healing phenomenon.

### 3.5. Microscopic Observation of Healing Effect

**Figure 8:**
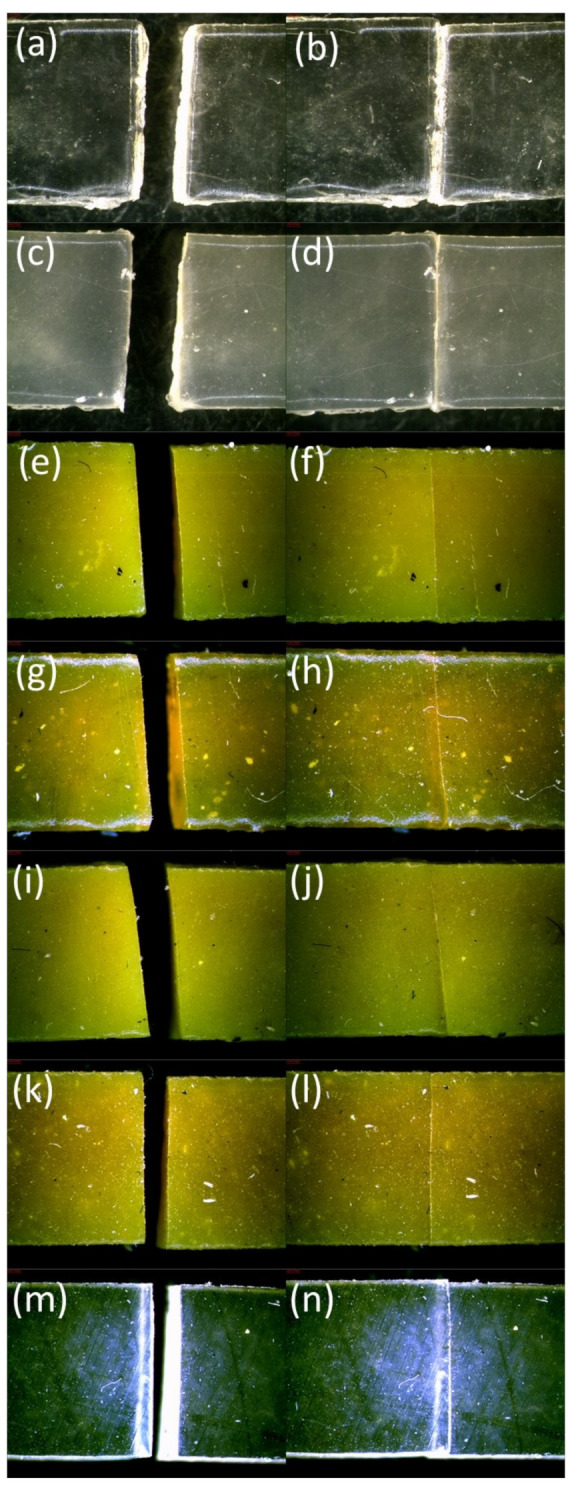
Pictures of samples after damage and after sudden healing of the damaged surfaces as a result of self-adhesion: pure ENR-50 sample after cutting (**a**) and after healing (**b**), ENR/silica15 composite after cutting (**c**) and after healing (**d**), ENR/quercetin2 sample after cutting (**e**) and after healing (**f**), ENR/quercetin2/silica15 composite after cutting (**g**) and after healing (**h**), ENR/quercetin4 material after cutting (**i**) and after healing (**j**), ENR/quercetin4/silica15 composite after cutting (**k**) and after healing (**l**), ENR/DCP2 composite after cutting (**m**) and after healing (**n**).

**Figure 9:**
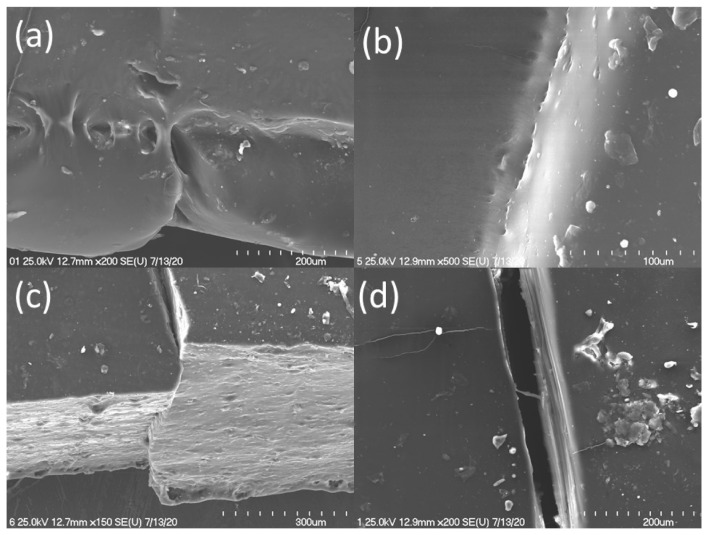
Picture of healed part of material observed in pure ENR (**a**), ENR/quercetin4 (**b**), ENR/quercetin4/silica15 (**c**) and ENR/DCP2 (**d**).

The self-healing phenomenon was also observed using an optical microscope and a scanning electron microscope. The optical microscope photos were taken to show the sudden self-adhesion after the connection of damaged sides together under a slight manual press. The photos of composites after the damage and after healing as a result of self-adhesion are presented below in Figure 8. According to the photos, the damaged surfaces of all tested composites were able to join to each other. The faulty part of the samples was observable as a scar, but in the cases of pure ENR and ENR with quercetin, such a defect was least visible. Based on the photos, ENR/quercetin2 and ENR/quercetin4 are the materials with the best self-adhesion. This is also confirmed by mechanical test results, where healing efficiency was calculated but other data show that the mechanical properties of these materials are not sufficient. To improve the mechanical properties, including tensile strength, silica was added, but this compound also caused a slight decrease in healing efficiency, which is visible in the pictures as well. To obtain a compromise between acceptable mechanical properties and satisfactory self-healing ability, silica must be added. The pure ENR and ENR/silica15 materials are transparent; thus, it is hard to show their self-adhesion in comparison to other samples in the same conditions. The self-adhesion of the tested materials is also confirmed by SEM photos introduced in Figure 9.

The best self-adhesion effect of the material is visible in pure ENR and ENR/quercetin materials. The totally mobile polymer chains are able to move and create new interactions between the cut surfaces. On the other hand, the samples made of ENR, quercetin and silica also reveal self-adhesion, where the polymer chains are partially mobile and are able to permeate the surfaces and form a new bonding. It must also be highlighted that the proper match is important and affects the healing efficiency. According to Figure 9c, the broken parts of the samples were not ideally aligned, which can give the impression of poor self-adhesion and can affect self-healing efficiency. Nevertheless, such a material reveals the compromise between the acceptable mechanical properties and satisfactory self-healing ability. In the case of the ENR/DCP2 composite, there are many free spaces, where surfaces are not connected. This material has the best mechanical properties, but the self-adhesion phenomenon is not acceptable.

## 4. Conclusions

The presented composites containing novel bio-based crosslinking systems based on pure quercetin and a combination of silica and quercetin reveal self-healing properties. The compromise between satisfactory tensile strength of above 3 MPa and the healing efficiency of around 40–45% was reached after adding silica combined with quercetin. The tensile strength of above 3 MPa might be considered to be low, but compared to the results of other self-healable biomaterials, for instance, composites of ENR and chitin nanocrystals containing hydrogen bonding supramolecular network with the tensile strength of 1.19 MPa [28], our materials seem to be promising. The self-healing phenomenon occurs as a result of self-adhesion of ENR combined with hydrogen bonding and other physical interactions formed between hydroxyl groups of quercetin and oxirane rings of ENR, between hydroxyl moieties of silica and epoxy groups of ENR and between hydroxyl groups of quercetin and silica. Such composites after 20 min of vulcanizing also revealed a higher crosslink density than ENR/silica or ENR/quercetin materials. It must be mentioned that biocomposites of ENR-50 containing quercetin and quercetin with silica are stiffer than conventionally cured elastomers with elongation at break of about 500–600% because of their rigid structure with aromatic rings of quercetin and strong interaction between silica, quercetin and ENR matrix. Nevertheless, the obtained results seem to be promising. A further improvement of the created composites is challenging but can result in obtaining self-healable long-term soft denture lining materials, because the selected components are safe for the oral cavity.

## Figures and Tables

**Table 1:** Compositions of studied ENR-50-based materials.

Components	Mass Ratio (phr)						
	ENR	ENR/ DCP2	ENR/ Silica15	ENR/ Quercetin2	ENR/ Quercetin4	ENR/ Quercetin2/ Silica15	ENR/ Quercetin4/ Silica15
ENR-50	100	100	100	100	100	100	100
DCP	-	2	-	-	-	-	-
quercetin	-	-	-	2	4	2	4
silica	-	-	15	-	-	15	15

phr—parts per hundred rubber.

**Table 2:** Values of the minimal torque (M_min_), torque after 20 min of curing (M_20_) and torque increase after 20 min of curing (ΔM_20_) of the same materials.

Composite	M_min_ (dNm)	M_20_ (dNm)	ΔM_20_ (dNm)
ENR	0.38	0.42	0.04
ENR/silica15	0.84	0.86	0.02
ENR/quercetin2	0.41	0.8	0.39
ENR/quercetin4	0.51	1.31	0.8
ENR/quercetin2/silica15	1.15	1.98	0.83
ENR/quercetin4/silica15	1.24	2.76	1.52
ENR/DCP2	0.38	4.34	3.96

## Data Availability

Data sharing is not applicable for this article.

## Supplementary Materials

The following are available online at https://www.mdpi.com/article/10.3390/ma14144028/s1, Table S1: Tensile strength (TS) results of pristine ENR-50-based composites and the same materials after 2 days, 4 days of self-healing without any external stimuli and after 4 days of self-healing with extra 20 min of heating, Table S2: Elongation at break (Eb) results of pristine ENR-50-based composites and the same materials after 2 days, 4 days of self-healing without any external stimuli and after 4 days of self-healing with extra 20 min of heating.
