# Supplementary material for: Self-Healable Biocomposites Crosslinked with a Combination of Silica and Quercetin
Source: Materials (Basel). 2021 Jul 19;14(14):4028. doi: 10.3390/ma14144028 (PMC8306683; doi:10.3390/ma14144028)
Supplement: Supplementary file 1 [file materials-14-04028-s001.zip › materials-1273097-supplementary.pdf]

materials

Supplementary Materials

Self-Healable Biocomposites Crosslinked with a Combination of Silica and Quercetin

Olga Olejnik [1], Anna Masek [1,*] and Małgorzata Iwona Szynkowska-Jóźwik [2]

[1] Institute of Polymer and Dye Technology, Faculty of Chemistry, Lodz University of Technology, Stefanowskiego 12/16, 90-924 Lodz, Poland; olejnik.olga@dokt.p.lodz.pl
[2] Institute of General and Ecological Chemistry, Faculty of Chemistry, Lodz University of Technology, Żeromskiego 116, 90-924 Lodz, Poland; malgorzata.szynkowska@p.lodz.pl
* Correspondence: anna.masek@p.lodz.pl

Table S1. Tensile strength (TS) results of pristine ENR-50-based composites and the same materials after 2 days, 4 days of self-healing without any external stimuli and after 4 days of self-healing with extra 20 minutes of heating.

Sample	$TS_{pristine}$ [MPa]	$TS_{after\ 2\ days\ of\ self\text{-}healing}$ [MPa]	$TS_{after\ 4\ days\ of\ self\text{-}healing}$ [MPa]	$TS_{after\ 4\ days\ of\ self\text{-}healing\ and\ 20\ min\ of\ heating}$ [MPa]
ENR	0.262 ± 0.014	0.21 ± 0.02	0.208 ± 0.014	-
ENR/quercetin22	0.61 ± 0.14	0.35 ± 0.07	0.38 ± 0.02	0.31 ± 0.08
ENR/quercetin4	1.04 ± 0.10	0.33 ± 0.08	0.36 ± 0.09	0.6 ± 0.5
ENR/silica15	2.3 ± 0.2	0.38 ± 0.04	0.52 ± 0.07	0.295 ± 0.012
ENR/quercetin2/silica15	3.3 ± 0.2	1.5 ± 0.6	1.3 ± 0.6	1.4 ± 0.3
ENR/quercetin4/silica15	3.8 ± 0.5	1.1 ± 0.5	1.4 ± 0.3	1.3 ± 0.5
ENR/DCP2	4.6 ± 0.2	0.4 ± 0.2	0.37 ± 0.03	0.6 ± 0.2

Table S2. Elongation at break (Eb) results of pristine ENR-50-based composites and the same materials after 2 days, 4 days of self-healing without any external stimuli and after 4 days of self-healing with extra 20 minutes of heating.

Sample	$Eb_{pristine}$ [%]	$Eb_{after\ 2\ days\ of\ self\text{-}healing}$ [%]	$Eb_{after\ 4\ days\ of\ self\text{-}healing}$ [%]	$Eb_{after\ 4\ days\ of\ self\text{-}healing\ and\ 20\ min\ of\ heating}$ [%]
ENR	1170 ± 90	80 ± 20	90 ± 30	-
ENR/quercetin22	800 ± 150	300 ± 200	370 ± 90	240 ± 150
ENR/quercetin4	770 ± 50	90 ± 60	130 ± 70	300 ± 300
ENR/silica15	910 ± 80	40 ± 20	150 ± 70	70 ± 60
ENR/quercetin2/silica15	620 ± 30	280 ± 140	250 ± 120	250 ± 90
ENR/quercetin4/silica15	510 ± 60	164 ± 104	200 ± 50	160 ± 80
ENR/DCP2	830 ± 20	50 ± 20	48 ± 8	140 ± 70
